# Planned Home Birth in Low-Risk Pregnancies in Spain: A Descriptive Study

**DOI:** 10.3390/ijerph18073784

**Published:** 2021-04-05

**Authors:** Trinidad M. Galera-Barbero, Gabriel Aguilera-Manrique

**Affiliations:** 1 Department of Nursing, Physiotherapy and Medicine, Faculty of Health Sciences, University of Almería, 04120 Almería, Spain; 2 Research Group for Health Sciences CST-451, University of Almería, 04120 Almería, Spain; gaguiler@ual.es

**Keywords:** low-risk pregnancy, maternal outcomes, midwife, newborn outcomes, obstetric interventions, planned home birth

## Abstract

Previous studies have shown that planned home birth in low-risk pregnancies is a generally safe option. However nowadays, only 0.5 percent of deliveries have been at home in Spain. This study sought to understand the characteristics of planned home births with qualified healthcare professionals in low-risk pregnancies and their results on maternal and neonatal health in the Balearic Islands. The study followed a retrospective descriptive design to investigate planned home births from 1989 to 2019 (*n* = 820). Sociodemographic data of women, healthcare professional intervention rates, and maternal/fetal morbidity/mortality results were collected. Statistical analysis of the results was performed using the IBM SPSS Version 25 software package. The results indicated that women with low-risk pregnancies who planned home births with a qualified midwife had a higher probability of spontaneous vaginal birth delivery and positive maternal health results. Furthermore, the risk of hospital transfer was low (10.7%) and the rate of prolonged breastfeeding (>1 year) was extremely high (99%). Moreover, the study showed that planned home births can be generally associated with fetal well-being. The conclusions and implications of this study are that planned home births in low-risk pregnancies attended by qualified midwives in the Balearic Islands achieve positive results in both maternal and newborn health, as well as low rates of obstetric intervention.

## 1. Introduction

Childbirth is one of the most important events in a woman’s life. During pregnancy the pregnant woman prepares psychologically and physiologically to give birth [1]. According to the World Health Organization (WHO) and the Spanish Ministry of Health and Social Policy, normal vaginal birth is defined as “spontaneous onset, low risk at the start of labor, remaining as such until delivery. The newborn is born spontaneously in the cephalic position between weeks 37 and 42. After giving birth, both the mother and the newborn must be in good condition” [2,3]. Furthermore, both parents should have the right to choose the place of birth with up-to-date information based on scientific evidence presented by qualified professionals [4].

Hospital births contribute 99% of deliveries in Spain. However, there is currently a large social and professional movement in favor of home births [5]. From 1996 (the last year recorded) to 2018, according to the Spanish National Statistics Institute (NSI), there were 26,175 home births in Spain, representing 0.36% of all births within the aforementioned time period. Most births at home happened in the region of Catalonia with 0.6%, followed by the Basque Country with almost 0.5% and the Balearic Islands with 0.43% (*n* = 1086) [6].

Currently, home birth remains a very rare choice amongst women in Spain. As it is not a delivery option covered by the National Health Service, pregnant women who wish to give birth at home must consult the services of private, independent midwives [7]. Consequently, the percentage of home births in Spain is low compared to other developed countries where this type of birthing option is offered within the public health system [8,9].

Home birth responds to the social and psychological needs of a pregnant woman in a personalized way whilst also allowing for complete autonomy over the partner a woman chooses whilst in labor [10,11]. The benefits and risks of childbirth within non-clinical settings have been widely debated in recent years as a consequence of the clear increase in the number of women who decide to give birth at home worldwide [12].

According to the results of the “Birthplace in England” study, as well as other research, women who plan home births have a lower risk of instrumental vaginal delivery, cesarean section, and a higher probability of spontaneous vaginal delivery than women who plan to deliver in hospitals [13,14,15,16,17].

Several studies have shown that home births are associated with a lower risk of maternal interventions compared to planned delivery in hospital in low-risk pregnant women [13,14,15,16,17,18,19,20]. Additionally, mothers who chose to deliver at home are more likely to breastfeed [21].

In terms of neonatal health outcomes, research offers conflicting arguments. According to Olsen and Clausen (2012) or Davies-Tuck (2018), a planned home birth is just as safe as a hospital delivery, whilst other studies associate it with adverse perinatal outcomes, such as low Apgar scores and increased risk of perinatal mortality [19,20,22,23,24,25,26,27,28,29,30].

According to the results of the “Birthplace in England” study and studies published by Hollowell [14], nulliparous women have a higher risk of adverse neonatal outcome (intrapartum fetal death, early neonatal death, neonatal encephalopathy, meconium aspiration syndrome and related specific injuries from birth, including brachial plexus injury) if they give birth at home compared to those who do so in hospital. However, no differences were observed between groups of babies born to multiparous women [14,15].

Few studies have evaluated the extent of transfer of women from a planned homebirth to hospital during labor or postpartum [31,32,33]. According to Blix’s study [32], the total proportion of hospital transfers varied from 9.9% to 31.9% [34]. Another investigation carried out in 2013 by Koettker concluded that the transfer rate was only 11% [35].

### Objective

The aim of this study was to identify the characteristics of planned home births with qualified healthcare professionals in low-risk pregnancies and their results in both maternal and neonatal health within the Balearic Islands, Spain from 1989 to 2019.

The specific objectives were:

- Specific objectives of the sociodemographic variables:
  - To explore the sociodemographic characteristics of women who choose this type of delivery.
- Specific objectives of clinical variables:
  - To know the characteristics of home birth, mode of birth, its length and its relationship with maternal age and parity, and, in relation to the need to be transferred to hospital, the causes, timing and factors on which the transfer depended and where these deliveries were finished (at home or in hospital).
  - To determine the rate of breastfeeding and participation in maternal education of women who planned a home delivery.
  - To identify the complications that birth at home entails for the mother and the newborn health and their association with parity, maternal age and gestational age.
  - To identify the maternal and neonatal mortality rate among deliveries that were planned to take place at home.
- Specific objectives of variables of professional intervention:
  - To highlight the obstetric interventions in childbirth and immediate postpartum planned at home, and its relationship to the length of delivery.

## 2. Materials and Methods

### 2.1. Design and Setting

The study was a retrospective descriptive quantitative study collecting data from planned home births in the Balearic Islands between 1989 and 2019.

The study sample was planned home births, even those that had to be transferred to hospital due to complications.

**Figure 1:**
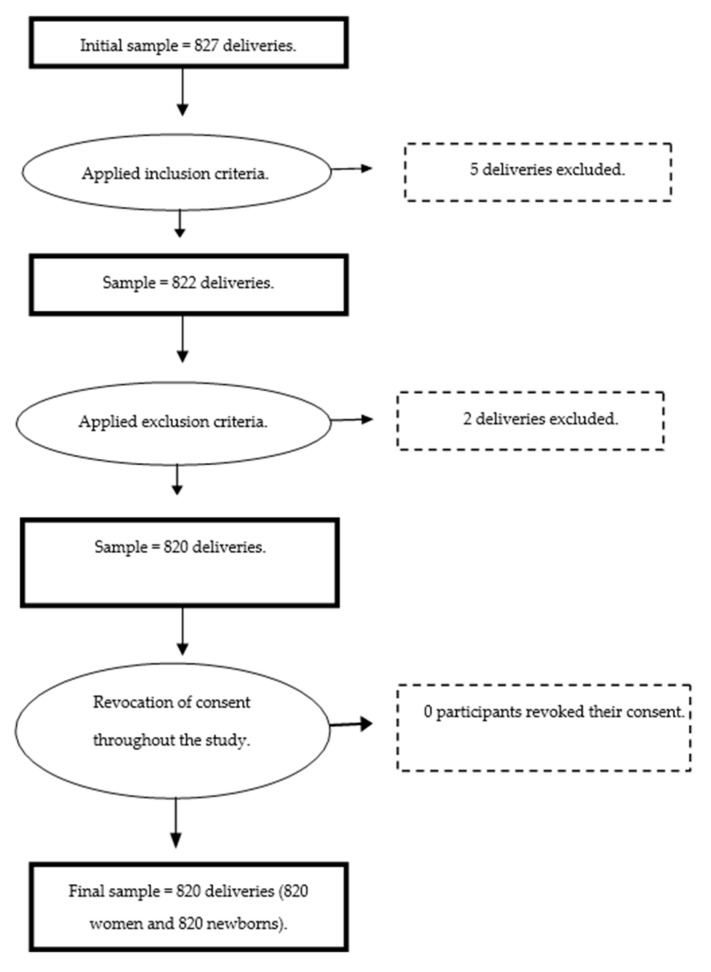
Flow chart of the study.

The initial sample of the study consisted of 827 planned home births assisted by private midwives. After the inclusion criteria were applied, 5 deliveries were discarded from the study (2 women with high risk pregnancies and 3 women without a minimum of four clinic visits before delivery) leaving a sample of 822 planned home births. Then, the exclusion criteria were applied and 2 more deliveries were excluded (2 women at more than 42 weeks of gestation). Finally no woman revoked her consent to participate in the study. The final sample was 820 planned home births, 820 women and 820 newborns (see Figure 1).

Eligibility criteria

In order to select the appropriate sample, a series of inclusion and exclusion criteria were proposed by researchers of the study.

Inclusion criteria:

○ Deliveries registered with standardized data collection form as established by the Guide to Home Birth Assistance [36].
○ Women who planned to give birth at home had to meet the criteria set forth in the Guide to Home Birth Assistance [36], to accept the request for accompaniment of home birth, which included the following:
  ○ The home birth plan is established before 28 weeks of gestation.
  ○ A minimum of four clinic visits before delivery.
  ○ The women must provide all the ultrasound and analytical controls and other necessary complementary tests that have been carried out.
  ○ Low-risk pregnancy, according to the “Guide to Clinical Practice for Childbirth Assistance” updated in 2018.
  ○ Maternal body mass index (BMI) ≤ 30 Kg/m^2^ at the beginning of pregnancy.
  ○ Clinical history without relevant complications.
  ○ Uncomplicated obstetric history (no previous cesarean sections).
  ○ No relevant signs or symptoms related to pregnancy complications, such as pre-eclampsia, intrauterine growth restriction, cholestasis… etc. [22,37].
  ○ The choice to give birth at home as an informed and free decision.

Exclusion criteria:

○ Multiple pregnancy.
○ Non-cephalic.
○ Start of delivery before week 37 or after week 42.
○ Qualification of the personnel assisting the delivery other than Obstetric-Gynecological nurses, or the absence of registration or civil liability insurance.
○ Distance between the home where delivery is intended and the reference hospital greater than 45 min by car.

### 2.2. Measurement Tools and Recruitment

The Balearic Islands have four hospitals providing obstetric care. From 1996 (last year recorded) to 2018, a total of 1086 women had home births in the Balearic Islands (0.43% of births are at home), with an average of 47 home births each year [6], being the third autonomous community with the highest number of home births. There are several groups of independent midwives who attending this type of delivery in the Balearic Islands.

As previously mentioned, home birth is not an option covered by the National Health Service in Spain, so all home birth services within the study were provided by midwives in private practices who were employed directly by the pregnant women. 

We made contact with midwives assisting home births as a means to collect data for the study. The data was registered using a predesigned data collection form (Supplementary Material File S1) as established by the Guide to Home Birth Assistance [36] by midwives and used routinely as a standard healthcare protocol in home births where all assisted delivery and postpartum was described.

According to the Guide to Home Birth Assistance [36] “in order to maintain standards of excellence in care for women and their family, it is essential to maintain updated records of all care offered and given. This will help maintain objectivity when it comes to offering attention to women, and also being able to transfer attention to other health professionals in a more fluid, complete and clear manner. In addition, audits should include, at least, the obstetric and perinatal results of childbirth, the derivations and/or transfers and the interventions that have been carried out.”

This standardized report details over 100 items regarding maternal characteristics, obstetric conditions and interventions, procedures and outcomes, maternal and perinatal mortality and morbidity and, finally, birth defects.

The researchers supervised all the data collected by these independent midwives and recorded all the variables of the study with this predesigned data collection form (Supplementary Material File S1).

### 2.3. Variables

The variables studied in this investigation were:

⮚ Sociodemographic variables:
  ∙ Maternal age.
  ∙ Parity.
  ∙ Gestation weeks.
  ∙ Place of residence.
  ∙ Mother’s education level.
⮚ Clinical variables:
  ∙ Characteristics of home birth (mode of birth and length of the deliveries).
  ∙ Maternal education.
  ∙ Breastfeeding.
  ∙ Maternal outcomes (transfer to hospital, perineal trauma, maternal complications…etc.).
  ∙ Newborn outcomes (weight, Apgar score after 5 min, newborn complications, intrapartum stillbirth, early neonatal mortality 0–7 days…etc.).
⮚ Variables of professional intervention:
  ∙ Intrapartum and postpartum medication.
  ∙ Kristeller maneuver (fundal pressure).
  ∙ Rupture of membranes (spontaneous or artificial) and moment.
  ∙ Bladder catheterization.

### 2.4. Data Analysis

All data were analyzed with the Statistical Package for Social Sciences (SPSS) Version 25 software (IBM Corporation, Armonk, NY, USA, 2025). The alpha level was set at 0.05 for all analyses. Simple descriptive statistics were used for analysis whilst Chi-square test and Fisher’s exact test were used to examine different variables.

First, a descriptive, one-dimensional analysis of a sample of all the variables to be studied was made. Measures of central tendency (mean) and measures of dispersion (standard deviation) of maternal age were obtained and the relative and the absolute frequencies of the remainder of the variables in the sample were calculated. Secondly, a two-dimensional analysis of the sample variables was carried out through the Chi-square test and Fisher’s exact test in order to identify the association between different variables (length of delivery related to perineal trauma, maternal age and parity; need to be transferred to the hospital related to parity; newborn health outcomes related to parity, and finally episiotomy related to dilation and expulsion time).

## 3. Results

### 3.1. Results of Sociodemographic Variables

The sample was composed of 820 planned home births. The age of the women who had planned to give birth at home was between 19 and 44 years old (M = 32.40, SD = 4.60). In total, 59% of the women gave birth at 38–40 weeks of gestation, followed by 28% of the participants who were between 40–41 weeks of gestation at the time of delivery. A total of 77% of the women already had a child, 36% were first-time mothers and 6% had already given birth to more than one child at the time of delivery. Additionally, 39% of women in the study had previously had home births. This amounted to a total of 320 participants who had previously given birth at home and 100% of them chose to repeat this method of delivery. Regarding the place of residence, 432 women lived in rural areas (52.7%) whilst 388 resided in the capital (47.3%) of the islands in which the research took place. With regards to mother’s educational level, 84% of the participants had completed university studies (see Table 1).

### 3.2. Results of Clinical Variables

In total, 97.1% (*n* = 796) of the deliveries were normal vaginal births, 2.4% (*n* = 20) were cesarean sections and 0.5% (*n* = 4) were instrumental births (with the use of a suction cup or forceps). A total of 257 deliveries lasted less than 6 h, from the first stage of the labor to delivery of placenta (afterbirth) (31.4%). Of those deliveries which lasted less than 6 h, 74.5% were of nulliparous women or those with a single child (*n* = 191) and 36% were between the ages of 30 and 34 years old (*n* = 93), (see Table 2).

Of the women, 76% (*n* = 623) attended maternal educational classes of some kind, and 43% (*n* = 353) had attended more than seven classes. A total of 99% of the mothers chose to breastfeed with 96.3% of cases continuing to breastfeed for a duration greater than one year (*n* = 790). None of the women suffered from puerperal mastitis or other complications (see Table 2).

In total, 10.7% of the women were transferred to hospital, the majority of whom were transferred during the first stage of labor (*n* = 88). The main reasons for transfer were due to the arrested cervical dilation or the arrested progress of the fetal head with 47% of the total (*n* = 41) followed by pain with 39% (*n* = 34). Of the transfers, 10% were due to fetal problems (seven cases during the first stage of labor due to fetal bradycardia and one case after expulsion due to an Apgar score < 7). A total of 2% of the transfers occurred due to maternal causes (two transfers due to intrapartum fever), (see Table 2).

In total, 75% of the pregnant women did not suffer any perineal trauma (*n* = 615) and only 0.5% of the women in the study had a perineal trauma of the third or fourth degree (*n* = 4). The maternal mortality rate was 0%. The maternal health outcomes are reflected below (see Table 3).

Regarding weight, 97.7% of newborns weighed between 2500 g and 4000 g (*n* = 801). More than 99.8% of the newborns obtained an Apgar score greater than 7 when collected five minutes after having been born (*n* = 818). There were no admissions to the neonatal intensive care unit. Likewise, the rate of intrapartum stillbirth and early neonatal mortality was 0% (see Table 4).

### 3.3. Results of Variables of Professional Intervention

Of the pregnant women who went on to give birth at home, 97% had their membranes broken for a period less than 18 h (*n* = 795). A total of 6.5% of the women who gave birth in their homes had no clear membranes fluid (see Table 2).

69% of mothers had spontaneous rupture of the membranes (*n* = 566). In total, 30% of midwife-induced artificial rupture of membranes were in the expulsion stage (*n* = 245). The Kristeller maneuver did not take place and only 15% of deliveries required episiotomies. In total, 96.3% of participants received no intrapartum or postpartum medication (*n* = 790). All obstetric interventions are presented in Table 5.

### 3.4. Other Results

Finally, for the purpose of exploring length of delivery and its relationship with maternal age and parity, and in relation to the need of hospitals transfers, the causes, timing and factors on which the transfer depended, the researchers made a two-dimensional analysis using Chi-square and Fisher’s exact test when the expected frequency was less than five in more than 20%.

It was found that the percentage of hospital transfers differed according to parity (χ^2^ (3, *N* = 820) = 81.85, *p* < 0.05). Nulliparous women were found to have a higher risk of transfer. Furthermore, neonatal health outcomes were discovered to be independent of parity. Nulliparous women did not have worse neonatal health outcomes than multiparous women (Table 6).

Additional findings suggested that the performance of episiotomy differed depending on the duration of the dilation (χ^2^ (3, *N* = 820) = 34.12, *p* < 0.05), but not the spontaneous trauma (χ^2^ (9, *N* = 820) = 10,63, *p* > 0.05). It was found that the longer this stage was, the greater the risk of episiotomy.

It was also found that both perineal trauma (χ^2^ (12, *N* = 820) = 35.28, *p* < 0.05) and episiotomy (χ^2^ (4, *N* = 820) = 53.97, *p* < 0.05) depended on the expulsion time during labor.

Finally, the duration of labor varied according to maternal age. Younger women had a longer dilation (χ^2^ (66, *N* = 820) = 132.97, *p* = 0.001) and expulsion (χ^2^ (88, *N* = 820) = 135.01, *p* = 0.001) than those of older age. Furthermore, the dilation duration depended on the parity (χ^2^ (9, *N* = 820) = 78.58, *p* = < 0.05). The same could be seen with the expulsion time (χ^2^ (12, *N* = 820) = 115.86, *p* < 0.05). The women with the highest parity experienced a shorter delivery compared to those who had not previously given birth.

## 4. Discussion

In Spain, very little research has been carried out on home births for a multitude of reasons, namely lack of means and funding and institutional support amongst others. However, outside of Spain, studies indicate that women who plan home births have a very low risk of instrumental vaginal delivery and cesarean section, and therefore a higher probability of spontaneous vaginal delivery. Additionally, findings in relation to outcomes are similar to with the investigations of Hollowell [14], Bolten [16] and Li [37].

With regard to hospital transfers, our study revealed a total proportion of transfers from home to hospital of 10.7%, being more frequent in nulliparous women than in multiparous women. This is supported by the studies of both Blix [32] and Koettker [35] who found the transfer rate from the home to hospital varied from 9.9% to 31.9%. Further similarities between our study and Blix’s research can be seen in the reasons given for hospital transfers-primarily due to slow progress of labor, followed by pain.

Our study indicated a favorable breastfeeding rate (BF) with 99% of mothers opting for exclusive BF during the first 6 months, and 96.3% breastfeeding for more than a year. This data coincides with the research carried out by Quigley in 2016 [21]. As far as the authors are aware, there are no published works on the rates of maternal education amongst women who decide to give birth at home so it is not possible to compare the results obtained in our study with others (76% of the participants attended some kind of maternal education and 43% attended more than seven classes).

In addition, home births were shown to have resulted in very low perineal trauma rates, either in the form of an episiotomy, any perineal trauma, or third or fourth degree perineal trauma. This is highlighted by the fact that more than 75% of the participating women suffered no perineal injury and only 15% of them had an episiotomy. Such findings coincide with the studies of The Birthplace in England Collaborative Group study [15], Scarf [17], Colacioppo [19] and Davies-Tuck [20].

Research on the safety of home births is mixed. This study indicated low risk of health complications in home births with a mortality rate in our sample of 0%, supporting the studies of Hollowell [14], Davies-Tuck [20] and Olsen and Clausen [24]. Furthermore, the neonatal health results achieved were very positive since the health deviations (Apgar at 5 min, need for ventilatory support, trauma, etc.) represented less than 0.2% of the newborns in the sample. Similar results were found within maternal health outcomes; less than 0.5% of the women in the study suffered from an infection or fever. Similar to the findings of Wax’s study [22], neonatal outcomes of planned home births revealed very low rates of low birth weight or macrosomia.

However, these data do not coincide with those obtained by Grünebaum [27], Hutton [26] and Davies-Tuck [20], who associate home birth with adverse perinatal outcomes, such as low Apgar scores and increased risk of perinatal mortality. Nor is there a significant difference between the neonatal results of nulliparous or multiparous mothers found within the Birthplace in England study or in the study developed by Hollowell in 2017 [14].

Finally, the research also showed that planned deliveries at home have a very low rate of maternal interventions since the Kristeller maneuver was not performed, artificial rupture of membranes (ARM) represented less than 35% of participants, 96% of women did not receive any type of medication during delivery or postpartum and only those that were transferred to the hospital for analgesic measures were probed. Women who chose home delivery used only 32% analgesia, with very low rates of epidural analgesia use (7%). These results can be complemented by the investigations of Fleming [10], Bolten [16], Colacioppo [19], Cheyney [38] and Jouhki [39].

Furthermore, as in the investigations of Olsen and Clausen [24], Givana Pimenta [40], Feeley [41], Henshall [42] and Naylor Smith [43], 100% of participants who had given birth at home in our studies wanted to repeat the experience of a home birth in future pregnancies.

### Strengths and Limitations

During the development of the study, the greatest challenge encountered was in finding and accessing an adequate sample.

Not only do home births continue to be a rare occurrence in Spain, but they are also exclusively carried out within the private sector. This meant that accessing the sample was difficult, which is why a retrospective descriptive study was chosen in order to have a larger sample. Nevertheless, the sample obtained is not as extensive as it should be. This made finding significant associations and generalizations from the data more challenging, since statistical tests tend to require a larger sample size to ensure a representative distribution of the population and thus to infer adequate results from the study.

Additionally, assistance with home births does not follow a definitive protocol, resulting in variability in care as each team of autonomous midwives acts differently and does not establish the same inclusion criteria for pregnant women. Another limitation of the studies was that they only incorporated those who, as we have previously mentioned, met the low-risk criteria established in the Guide to Home Birth Assistance [36]. To increase the sample size, the researchers chose a retrospective study design for the purpose of incorporating more mothers who had given birth at home. Because of that, the data were collected years before this study was designed and no training could be given to the midwives apart from the standardized data collection procedure. However, in order to select the appropriate sample, a series of inclusion and exclusion criteria were applied by the researchers, for instance all deliveries must be registered with a predesigned data collection form as established by the Guide to Home Birth Assistance [36].

A final drawback of a descriptive studies approach is that it does not allow for an exploration of the causal association between variables and thus the subsequent results can be limited.

For this reason, it is our purpose to develop new research that includes a greater number of home births and to compare the results obtained with hospital births, in order to determine the risk and benefits of home birth in Spain.

## 5. Conclusions

To identify the characteristics of planned home births with qualified healthcare professionals in low-risk pregnancies and their results in both maternal and neonatal health, we explored the characteristics of home birth and the women who choose this type of delivery. An average age of 32 and university-level of education was found amongst women planning a home birth in the Balearic Islands. Parity and place of residence were not significant characteristics. Home delivery was associated with a high probability of vaginal birth delivery and a low transfer rate, especially during dilation. However, nulliparous women had a higher risk of transfer.

Secondly, the rate of breastfeeding and participation in maternal education of women who have planned given birth at home were investigated. The results showed that a high rate of breastfeeding and participation in maternal education were found.

Then, maternal and neonatal health outcomes were evaluated. The maternal health results obtained in the study were very positive for both nulliparous and multiparous women. Similarly, within neonatal health results, in most cases high Apgar scores and low probability of morbidity and mortality were achieved regardless of parity.

Finally, the obstetric interventions in childbirth and immediate postpartum planned at home were explored, as was its relationship to the length of delivery. Planned home birth offered low obstetric intervention assistance both during delivery and postpartum.

Despite such findings, further studies are needed to assess the maternal and newborn health outcomes of home birth with qualified healthcare.

## Figures and Tables

**Table 1:** Descriptive statistics of the sociodemographic variables of the sample.

	*N*	%
**Maternal age**		
<20 years	1.64	0.2%
20–24	28.7	3.5%
25–29	196.8	24%
30–34	324.7	39.6%
35–39	216.4	26.4%
>40	51.6	6.3%
**Place of residence**		
Metropolis	388	47.3%
Rural environment	432	52.7%
**Mother’s education level**		
Primary studies	0	0%
Secondaries studies	131.2	16%
University studies	688.8	84%
**Parity**		
Nulliparity	296	36.1%
Multiparity	524	63.9%
Previous home birth	319.8	39%
**Gestation weeks**		
<38 weeks	38	5%
38–39 + 6	426	59%
40–40 + 6	232	28%
41–41 + 6	62	8%
>42 weeks	3	<0.5%

**Table 2:** Descriptive statics of the clinical variables.

	Nulliparous Women (*n* = 296)	Multiparous Women (*n* = 524)	Total (*n* = 820)
**Mode of birth**			
*Normal vaginal birth*	94.3% (*n* = 279)	98.7% (*n* = 517)	97.1% (*n* = 796)
*Instrumental birth*	0.3% (*n* =1)	0.6% (*n* = 3)	0.5% (*n* = 4)
*Cesarean section*	5.4% (*n* = 16)	0.7% (*n* = 4)	2.4% (*n* = 20)
**Delivery time**			
*Dilation time*	M = <5 h	M = <5 h	M = <5 h
*Expulsion time*	M = 10–30 min	M = 10–30 min	M = 10–30 min
*Afterbirth time*	M = <30 min	M = <30 min	M = <30 min
**Attendance of the participants to Maternal Education**	86.8% (*n* = 257)	70.2% (*n* = 368)	76% (*n* = 623)
**Newborn feeding**			
*Breastfeeding*	99.3% (*n* = 294)	99% (*n* = 519)	99.1% (*n* = 813)
*Artificial lactation*	0.7% (*n* = 2)	0.4% (*n* = 2)	0.4% (*n* = 3)
*Mixed lactation*	0%	0.6% (*n* = 3)	0.5% (*n* = 4)
*Duration of breastfeeding*	96.6% > 1 year (*n* = 286)	95.6% (*n* = 501)	95.4 > 1 year (*n* = 782)
*Mastitis or another complication during breastfeeding*	0%	0%	0%
**Transfer to hospital**	23.6% (*n* = 70)	3.4% (*n* =18)	10.7% (*n* = 88)
*Transfer during delivery*	98.6% (*n* = 69)	88.9(*n* = 16)	96.6% (*n* = 85)
*Transfer post delivery*	1.4% (*n* = 1)	10.1% (*n* = 2)	3.4% (*n* = 3)
**≥18 h Amniotic sac broken**	4.1% (*n* = 12)	2.3% (*n* = 12)	3% (*n* = 24)
**Not clear amniotic fluid**	6.8% (*n* = 20)	6.3% (*n* = 33)	6.5% (*n* = 53)

**Table 3:** Maternal health outcomes.

Maternal Health Outcomes	Nulliparous Women	Multiparous Women	Total
**Fever**	0%	0.6% (*n* = 3)	0.4%
**Haemorrhage**	1.6% (*n* =5)	2.1% (*n* = 11)	2%
**Infections**	0.3% (*n* = 1)	0%	0.1%
**Perineal trauma**	27.4% (*n* =81)	23.3% (*n* = 122)	25%
*Perineal trauma of the 1st degree*	77.8% (*n* = 63)	47.5% (*n* = 58)	14.8%
*Perineal trauma of the 2nd degree*	21% (*n* = 17)	50% (*n* = 61)	9.5%
*Perineal trauma of the 3rd or 4th degree*	1.2% (*n* = 1)	2.5% (*n* = 3)	0.5%
**Mortality**	0%	0%	0%

**Table 4:** Newborn health outcomes.

Newborn Health Outcomes	Nulliparous Women	Multiparous Women	Total
**Apgar score < 7 after 5 min**	0.3% (*n* = 1)	0.2% (*n* = 1)	0.2% (*n* = 2)
**Trauma**	0%	0.4% (*n* = 2)	0.2% (*n* = 2)
**Caput**	2% (*n* = 6)	0.7% (*n* = 4)	1.2% (*n* = 10)
**Meconium Aspiration Syndrome**	0%	0.2% (*n* = 1)	0.1% (*n* = 1)
**Ventilatory support**	0%	0.2% (*n* = 1)	0.1%
**Intrapartum stillbirth**	0%	0%	0%
**Early neonatal mortality 0–7 days**	0%	0%	0%

**Table 5:** Obstetrics interventions.

Professional Intervention	Nulliparous Women	Multiparous Women	Total
**Artificial rupture of membranes**	34.1% (*n* = 101)	29.3% (*n* = 154)	31% (*n* = 254)
**Spontaneous birth**	99.7% (*n* = 295)	99.8% (*n* = 523)	99.7% (*n* = 818)
**Intrapartum and postpartum medication**	7.8% (*n* = 23)	1.3% (*n* = 7)	3.7% (*n* = 30)
**Kristeller maneuver**	0%	0%	0%
**Episiotomy**	19.6% (*n* = 58)	12.4% (*n* = 65)	15% (*n* = 123)
**Bladder catheterization**	13.5% (*n* = 40)	1.7% (*n* = 9)	6% (*n* = 49)
**Anesthesia**	39.9% (*n* = 118)	28.2% (*n* = 148)	32.4% *(n* = 266)
**Delayed umbilical cord clamping**	100% (*n* = 296)	100% (*n* = 524)	100% (*n* = 820)

**Table 6:** Chi- square results of newborn health outcomes.

Variables	Chi-Square
*Need of ventilatory support-Parity*	χ^2^ (3, *N* = 820) = 1.42, *p* = 0.649
*Fetal secretions aspiration syndrome-Parity*	χ^2^ (3, *N* = 820) = 4.29, *p* = 0.176
*Meconium aspiration syndrome-Parity*	χ^2^ (3, *N* = 820) = 1.42, *p* = 0.649
*Neonatal death-Parity*	χ^2^ = 0
*Intrapartum fetal death-Parity*	χ^2^ = 0
*Caput-Parity*	χ^2^ (3, *N* = 820) = 3.87, *p* = 0.318
*Trauma-Parity*	χ^2^ (3, *N* = 820) = 2.21, *p* = 0.551
*Apgar < 7 at 5 min.-Parity*	χ^2^ (6, *N* = 820) = 3.19, *p* = 0.771

## Data Availability

The datasets generated and/or analyzed during the study are not publically available due to being obtained from a third party. The data were provided by midwives in private practice, but are available from the corresponding author on reasonable request.

## Supplementary Materials

The following are available online at https://www.mdpi.com/article/10.3390/ijerph18073784/s1, File S1: Supplemental Information: Predesigned data collection sheet.
