# Supplementary material for: Planned Home Birth in Low-Risk Pregnancies in Spain: A Descriptive Study
Source: Int J Environ Res Public Health. 2021 Apr 5;18(7):3784. doi: 10.3390/ijerph18073784 (PMC8038591; doi:10.3390/ijerph18073784)
Supplement: Supplementary file 1 [file ijerph-18-03784-s001.pdf]

1. Supplemental Information: Predesigned data collection sheet.

Delivery date: Age: Parity: Gestation weeks: Place of residence: Level of studies:

Was the transfer to the hospital necessary? When? And why?

How was the beginning of the delivery?

Dilation duration: <5 hours / 5 - 10 hours / 10 - 15 hours /> 15 hours

Expulsion duration: 5 - 10 minutes / 10 - 30 minutes / 30 mint. - 1 hour / 1 - 2 hours /> 2 hours.

Afterbirth duration: <30 minutes / 30 mint. - 1 hour / 1 - 2 hours /> 2 hours.

Type of delivery: Spontaneous / Pharmacological.

Amniotic bag rupture: Spontaneous / Artificial. When? Dilation / Expulsive.

> 18 hours of broken amniotic bag? Yes / No. Color of Amniotic Fluid? Clear / Tinted / Other.

Umbilical cord clamping time: 0 - 15 seconds / 15 - 30 seconds /> 30 seconds

Nuchal cords? None / 1/2 /> 2.

Did you attend maternal education? No / Yes, at 1 - 3 sessions / Yes, at 4-6 sessions / Yes, at> 7 sessions.

Have you been given intrapartum medication? Which?

Have you been given postpartum medication? Which?

Did they do the Kristeller Maneuver? Yes / No.

Did you have a perineal trauma of I, II, III or IV degree?

Did you have an episiotomy? Yes / No.

Did you have anesthesia? No / Yes, local anesthesia / Yes, Epidural anesthesia / Yes, other.

Did you have a bladder catheter? Yes / No.

Mode of Birth: Eutocic /Vaginal assitance/ Cesarean section.

Did you have intrapartum or postpartum fever? Yes / No. Was there postpartum hemorrhage? Yes / No Why?

Did you have an infection? Yes / No.

Newborn feeding: Breastfeeding / Artificial laction / Mixed lactation.

Duration of breastfeeding? <3 months / 3 - 6 months / 6 - 1 year /> 1 years.

Newborn weight: <2,500gr / 2,500 - 4,000gr /> 4,000gr.

Apgar score after 5 minutes: 9 - 10 points / <7 points / <4 points / 0 points.

Did the newborn suffer any trauma? Yes /No Which?

Did Caput have the newborn? Yes / No. Intrapartum stillbirth? Yes / No.

Early neonatal mortality 0–7 days? Yes / No. Maternal mortality in childbirth or Postpartum? Yes /No.

Did the newborn suffer from Meconial Aspiration Syndrome? Yes / No.

Did the newborn need aspiration of secretions? Yes / No.

Did the newborn need ventilatory support? Yes / No.
